# Anti‐CLL1‐based CAR T‐cells with 4‐1‐BB or CD28/CD27 stimulatory domains in treating childhood refractory/relapsed acute myeloid leukemia

**DOI:** 10.1002/cam4.5916

**Published:** 2023-04-09

**Authors:** Kunlin Pei, Haoyu Xu, Pengfei Wang, Wening Gan, Zhengbin Hu, Xiaoling Su, Hui Zhang, Yingyi He

**Affiliations:** ^1^ Department of Hematology/Oncology, Guangzhou Women and Children's Medical Center Guangzhou Medical University Guangzhou 510623 Guangdong China; ^2^ Guangzhou Medical University Guangzhou 511495 Guangdong China

**Keywords:** 4‐1‐BB, acute myeloid leukemia, anti‐CLL1 CAR T cells, CD28/CD27, refractory/relapsed

## Abstract

**Background:**

Though the efficacy of anti C‐type lectin‐like molecule‐1 (CLL1) CAR T‐cells in refractory/relapsed acute myeloid leukemia (R/R‐AML) have been occasionally reported, the influence of co‐stimulatory domain CAR T‐cells is not investigated so far.

**Method:**

Seven R/R‐AML children treated with anti‐CLL1 CAR T‐cells were enrolled onto this preliminary comparison study. Among these seven patients, four received CD28/CD27‐based CAR T‐cells therapy, and three received 4‐1BB‐based CAR T‐cells therapy.

**Result:**

The overall response rates were 75% and 66.7% in CD28/CD27 and 4‐1BB group respectively. All patients experienced grade 1 to 2 cytokine release syndrome, with only one patient experiencing grade 2 immune effector cell‐associated neurotoxicity syndrome. The maximum CAR T‐cells durations were 156 and 274 days for CD28/CD27 group and 4‐1BB group respectively. The 1‐yr overall survival rate was 57.1%.

**Conclusions:**

A preliminary similar efficacy/safety index was observed in anti‐CLL1‐based CAR T‐cells with 4‐1BB or CD28/CD27 co‐stimulatory elements in treating pediatric R/R‐AML.

## Graphical abstract

This preliminary report demonstrates that CD28/CD27‐ and 4‐1‐BB‐equipped CAR T‐cells have comparable effectiveness and safety indices in treating children with relapsed/refractoryacute myeloid leukemia.

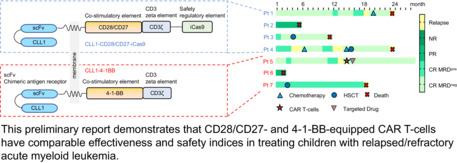

Nowadays, nearly 50% of pediatric AML patients in developed countries would experience relapse and finally succumb to disease progression, with 5‐year event‐free survival rate less than 60%, which is even worse in developing countries. Therefore, novel therapeutic strategies are highly needed for relapsed/refractory acute myeloid leukemia (R/R‐AML) therapy.

Though multiple agents have been developed, their clinical translation remains obscure due to the benefit to specific AML subpopulation, systemic toxicities, and acquired drug resistance later on,^1^ particularly for the pediatric population. Chimeric antigen receptor (CAR) T‐cells therapy, an adoptive cell therapy, is becoming an attractive candidate. Attractively, anti‐CLL1‐based CAR T‐cells have been proven clinically safe and well responded in R/R‐AML,^2^, ^3^, ^4^ holding the promise as a new therapy for R/R‐AML. However, the clinical translation of CAR T‐cells is still facing a lot of challenges, including lack of tumor‐specific CAR T target antigens, T‐cell aplasia, malignant T‐cell contamination, and fratricide in T‐ALL patients, antigen‐shift in AML.^5^, ^6^ Thus, further studies are warranted to achieve a better treatment outcome.

Co‐stimulatory domains play an indispensable role, that is, CAR T‐cells activation, persistence, and its targeted cytotoxicity.^7^, ^8^, ^9^ However, the impact of different co‐stimulatory elements on anti‐CLL1‐based CAR T‐cells in treating AML has not been investigated so far. To this end, we here preliminarily compared the safety and efficacy index of anti‐CLL1‐based CAR T‐cells between those equipped with 4‐1‐BB domain and CD27/CD28 domain in treating children with R/R‐AML.

All the medical data were collected, and critically reviewed for all these enrolled R/R‐AML patients receiving anti‐CLL1‐based CAR T‐cells therapy from September 2018 to December 2020. Prior to anti‐CLL1 CAR T‐cells infusion, all patients received lymphodepletion chemotherapy (cyclophosphamide, 500–900 mg/m^2^/day, from Day 4 to Day −1; fludarabine, 25 mg/m^2^/day, from Day −2 to Day −1 depending on the AML burden).^2^, ^3^, ^10^ NCCN guidelines version 3.0 on AML was used to assess the treatment efficacy.^11^ Side effect was defined as an unrelated pharmacological effect occurring within 4 weeks after CAR T‐cells therapy. The adverse effects of anti‐CLL1 CAR T‐cells therapy were evaluated and graded using the CTCAE 5.0 criteria. In the meanwhile, the CAR T‐cells therapy‐related toxicities, for example, cytokine release syndrome (CRS), immune effector cell‐associated neurotoxicity syndrome (ICANS), hemophagocytic lymphohistiocytosis (HLH), and macrophage activation syndrome (MAS), were evaluated using the management system proposed by Santomasso et al.^12^, ^13^

Seven patients were included in this prospective study; four were treated with CD28/CD27‐equipped anti‐CLL1‐based CAR T‐cells while three were treated with 4‐1‐BB‐equipped anti‐CLL1‐based CAR T‐cells (Figure 1A and Table 1). The illustration of CAR constructs is shown in Figure 1B. The median age of patients was 8.4 years (range, 5.8–13.5 years), The median white blood cells (WBC) counts were 1.0 × 10^9^/L (range 0.4–1.5) and 0.4 × 10^9^/L (range 0.1–0.7) in the CD28/CD27 and 4‐1‐BB groups, respectively. The minimal leukemic burden varied from 0.04% to 96.4% as reflected by flow cytometry. These patients received anti‐CLL1‐based CAR T‐cells varying from 0.94 to 1.98 × 10^6^/kg body weight after prior lymphodepletion therapy (Figure 1C).

**FIGURE 1:**
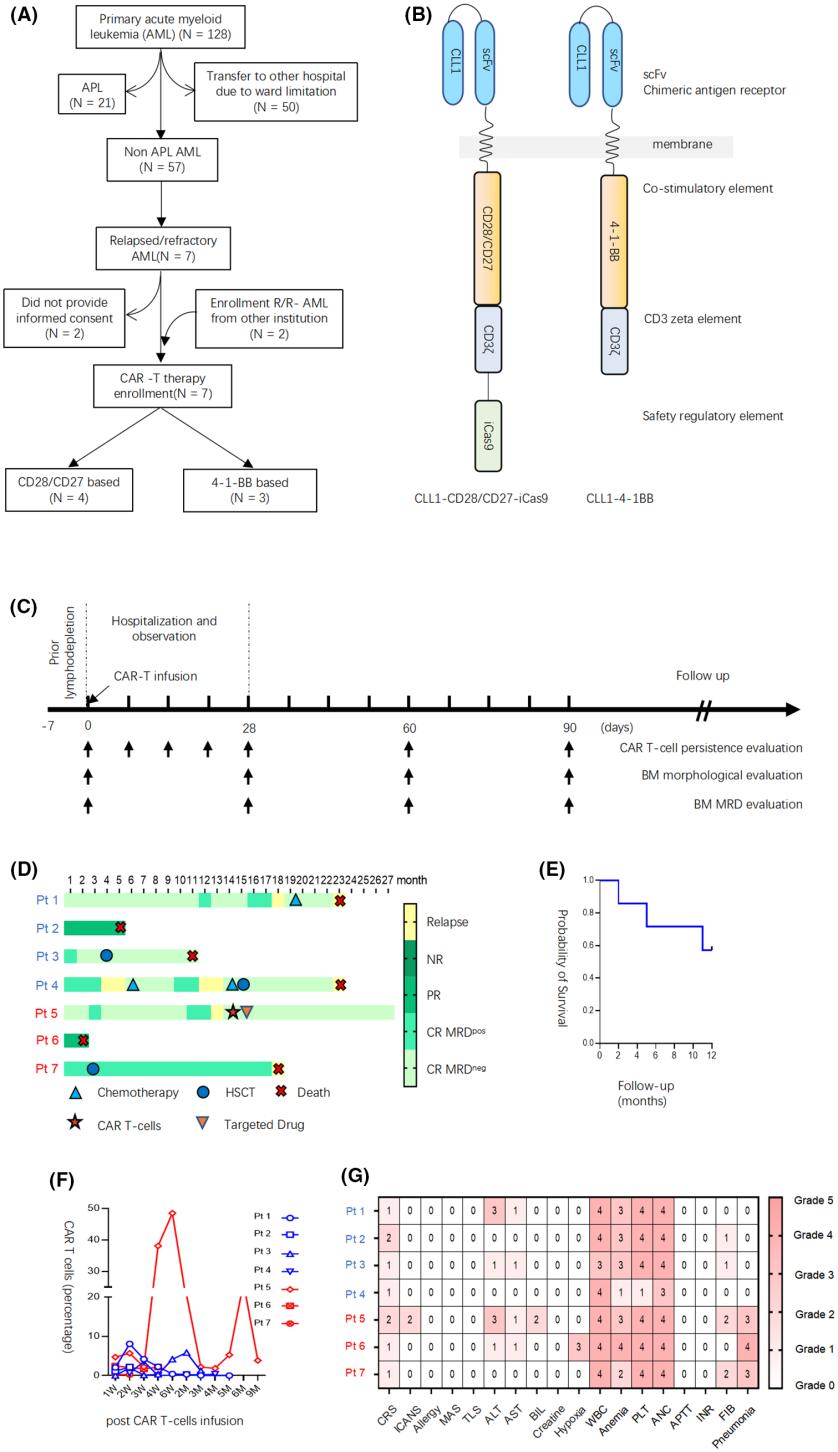
Treatment response and related adverse events evaluation. (A) The process of patient enrolment. (B) The illustration of CAR constructs. The light blue oval represents scFv chimeric antigen receptor, the light orange box represents co‐stimulatory domains, the light purple box represents CD3 zeta element, and the light green box represents inducible Cas9 domain. (C) The treatment and follow‐up plan for this study. The black arrow used in panel B represented the time point that we performed the clinical evaluation. (D) The treatment response to anti‐CLL1‐based CAR T‐cells therapy is plotted against treatment duration time (months). The darker the blue is, the worse response it is. (E) The follow‐up plot of seven enrolled patients. The red cross represents death event, the solid dark blue circle represents HSCT, the solid light blue triangle represents further chemotherapy, the red pentagram represents CAR T‐cells therapy and the orange triangle represents targeted drug therapy. (F) The 1‐year overall survival among these seven enrolled patients. (G) The toxicity grade for these seven patients is plotted against each kind of related adverse event. The darker the red is, the higher grade of toxicity it is. The number in the grid exactly reflects the toxicity grade. The blue word represents CAR T‐cells equipped with CD28/CD27 co‐stimulatory elements, and the red word represents CAR T‐cells equipped with 4‐1‐BB co‐stimulatory elements. CRS, cytokine release syndrome; ICANS, immune effector cell‐associated neurotoxicity syndrome; MAS, macrophage activation syndrome; TLS, tumor lysis syndrome; ALT, alanine aminotransferase; AST, aspartate aminotransferase; BIL, bilirubin; WBC, white blood cell; PLT, platelet; ANC, absolute neutrophil count; APTT, activated partial thromboplastin time; INR, international normalized ratio; FIB, fibrinogen.

**TABLE 1:** Clinical characteristics of enrolled R/R‐AML patients.

Patient no.	#1	#2	#3	#4	#5	#6	#7
Age/Gender	9.6/F	8.4/F	7.3/M	5.8/M	8.2/M	13.5/F	12.1/M
WBC_Dx (×10^9^/L)	2.2	66.6	10.2	19.1	2	N.A	N.A
FAB subtype	NOS	MDS‐AML	M2a	M5a	M6	M2	M2a
FISH	MLLr	−7	N.A	Normal	N.A	Normal	Normal
Karyotype	46, XX[10]^10^	45, XX, −7[9]^9^	46, XY [20]	46, XY[10]^10^	N.A	45, X,‐X,inv(11)(p15q23),del(15)(q22)[17]^17^	48, XY,+?Y,+8 [20]
Fusion gene	*KMT2A‐CREBBP*	*EVI1*	*NUP98‐NSD1*	*KMT2A‐MLLT10* *MLLT10‐UBE4A*	N.A	NUP98‐DDX10	N.A
Mutations	*WT1* ^*S381fs*^	*NRAS* ^*Q61H*^	*FLT3‐ITD*	*NRAS* ^*G12D*^	*WT1* ^*R458P*^	*KRAS* ^*G13C*^	*IDH1* ^*R132C*^
	*RUNX1* ^*R204P*^	*NRAS* ^*S65T*^	*WT1* ^*P377fs*^	*PTPN11* ^*T73I*^		*WT1* ^*H469D*^	*STAT5B* ^*N642H*^
	*TCF12* ^*P73fs*^	*NRAS* ^*R68T*^	*CEBPA* ^*Q83fs*^	*SETD2* ^*R1407fs*^		*WT1* ^*H465R*^	*PHF6* ^*M46fs*^
		*RUNX1* ^*D198G*^	*PTPN11* ^*A72V*^			*ETV6* ^*R105fs*^	
		*BRAF* ^*E501K*^	*BRCA1* ^*I986fs*^			*RUNX1* ^*D137H*^	
			*MYC* ^*P74Q*^			*GATA2* ^*L375I*^	
			*LRP2* ^*D1829E*^			*KDM5C* ^*A887fs*^	
WBC_pre‐CAR T (×10^9^/L)	0.9	1.1	0.4	1.5	0.7	0.1	0.1
T cells (cells/μL)	9.63	12.66	14.26	1161.89	862.14	224.55	707.91
% T cells	90.76	80.95	84.88	88.36	87.49	81.19	79.5
B cells (cells/μL)	0.58	1.83	0.19	8.90	72.74	0.79	2.71
% B cells	5.47	11.70	1.26	0.68	7.38	0.29	0.30
NK cell (cells/μL)	0.41	1.15	0.58	144.09	5051	51.22	179.82
% NK cell	3.86	7.35	3.86	10.96	5.13	18.52	20.19
% Blast pre‐CAR T	19.5	9.5	6.5	1.0	9.0	71.5	23.0
% MRD pre‐CAR T	17.6	3.14	6.84	0.04	14.19	96.4	3.5
CAR specificity	CLL1	CLL1, Lewis‐Y	CLL1, CD33, CD38	CLL1, CD33	CLL1	CLL1	CLL1
Co‐stimulatory domain	CD28/CD27	CD28/CD27	CD28/CD27	CD28/CD27	4‐1‐BB	4‐1‐BB	4‐1‐BB
CAR T‐cell dose (×10^6^/kg)	1.49	1.00	1.98	1.98	1.03	0.94	0.94
% CAR transduction efficiency	95.05	75.90	13.54	78.20	35.79	38.30	64.96
Lansky score	80	80	90	80	70	60	80
Viral status (pre‐CAR T)	No viral infection	No viral infection	No viral infection	No viral infection	HSV	N.A.	No viral infection
Viral status (post‐CAR T)	No viral infection	No viral infection	CMV in HSCT	No viral infection	HSV	N.A.	N.A.
CRS grade	1	2	1	1	2	1	1
ICANS grade	0	0	0	0	2	0	0
Liver impairment grade	3	0	1	0	3	1	0
Follow‐up time (months)	23	5	11	23	27	2	18
MRD marker	CD15	CD123	CD123	CD123	CD123	CD133	CD123
Current status	Death	Death	Death	Death	Alive	Death	Death
Cause of death	Relapse	Progression	GVHD	Progression	N.A	Progression	Relapse

The early treatment response was monthly evaluated in the first 3 months. Five patients achieved complete remission (CR) with three patients negative and two patients positive for minimal residual disease (MRD) (Figure 1D). The overall response rate was 75% (3/4) and 67% (2/3) in the CD28/CD27 and 4‐1‐BB groups, respectively (Figure 1D). Meanwhile, the 1‐year survival was 57.1% among these seven enrollment cases (Figure 1E). In this report, we monitored the level of anti‐CLL1 CAR T‐cells to evaluate the association between CAR T‐cells efficacy and CAR T‐cells expansion. The CAR T‐cells of three patients (patients 1, 3, and 5) were successfully expanded and persisted with an average of 6 months (Figure 1F). Interestingly, all these three patients achieved CR with MRD negativity. At the time of submission, the latest follow‐up via telephone for all these seven patients was completed. The follow‐up duration varied from 2 to 27 months among these seven patients. As illustrated in Figure 1D, patients 2 and 6 died of disease progression after 5 and 2 months of anti‐CLL1 therapy, respectively. For patient 1, CR was achieved and maintained for 18 months, with MRD negative for 12 months. Unfortunately, patient 1 experienced relapse 18 months after CAR T‐cells therapy and received chemotherapy again. Within ~6 months of the second CR, patient 1 succumbed to leukemia recurrence. Patient 3 received haploidentical 7/10 HLA‐matched allogeneic hematopoietic stem cell transplantation (allo‐HSCT) 2 months after anti‐CLL1 CAR T‐cells infusion and remained negative for AML blast for 8 months. Unluckily, he died of gastrointestinal graft‐versus‐host disease (GVHD) and thrombotic microangiopathy 9 months after the transplant. Patient 4 did not respond to CAR T‐cells therapy, presented with persistent MRD and experienced relapse 4 months later. He received a combination of hypomethylating agents‐based chemotherapy and venetoclax. Though the disease was improved again for 6 months long, he eventually experienced relapse again 12 months after CAR T‐cells therapy. Even though proceeding to allo‐HSCT therapy, patient 4 died of disease progression eventually. Patient 7 received allo‐HSCT 3 months after CAR T‐cells therapy and remained morphologic leukemia‐free status for 14 months. Unfortunately, patient 7 died of relapse after 18 months of CAR T‐cells therapy. At the time of manuscript submission, only patient 5 is still alive, achieving 12 months of CR after CAR T‐cells therapy. Without proceeding to allo‐HSCT, patient 5 experienced relapsed at the 13th month and was maintained on venetoclax treatment after receiving a second autologous anti‐CLL1 CAR T‐cells therapy, and remained leukemia‐free status.

To address the safety issue, we critically reviewed the clinical data and presented the adverse events (AEs) in Figure 1G. All seven patients experienced Grade 1 or 2 CRS manifested by fever without evidence of infection during the first month as reflected by increased serum IL‐6 level (Figure S1). Among these patients, patient 5 experienced Grade 2 ICANS as presented with transientl attention loss, speech impediment, and tremors. Two patients (patients 1 and 5) had Grade 3 impaired liver function as reflected by increased alanine aminotransferase level, and patient 5 was accompanied by Grade 2 increased bilirubin level. It is well established that CLL1 is expressed on lung parenchyma, we observed Grade 3 pneumonia without hypoxia in patients treated with 4‐1‐BB anti‐CLL1‐based CAR T‐cells but not in the CD28/CD27 group, suggesting the potential role of 4‐1‐BB in CLL1‐targetin‐related lung injury. Further studies are warranted to determine whether anti‐CLL1‐based CAR T‐cells impair lung parenchyma. According to the hematologic toxicities, all these seven patients developed Grade 3 or 4 WBC counts decrease and neutropenia, five of them developed Grade 3 or 4 anemia, and six of them developed thrombocytopenia. The monocytopenia was observed in most responded patients, suggesting the potential role of monocyte ablation in reflecting anti‐CLL1 CAR T‐cells therapy. (Figure 1G, Figure S2). No allergy, MAS, and HLHs were observed.

In the present study, we summarized the treatment outcome of seven children with R/R‐AML treated with anti‐CLL1‐based CAR T‐cell therapy, aiming to shed new light on the translation of anti‐CD19 CAR T‐cell therapy in B‐ALL into R/R‐AML treatment. The 1‐year survival rate was 57.1% among these seven cases, while the 1‐year survival rate was 80% if poorly responded patients were excluded (Figure S3). Only Grades 1 or 2 CRS were observed and successfully managed. Though CLL1 is constitutively expressed on lung parenchyma, we observed that all three patients receiving 4‐1‐BB anti‐CLL1‐based CAR T‐cells developed pneumonia in the first month of CAR T‐cells therapy, suggesting the potential role of 4‐1‐BB in CLL1‐targeting related lung injury. Further studies are warranted to determine whether anti‐CLL1‐based CAR T‐cells impair lung parenchyma.

Recent studies have demonstrated that CD28‐based CAR T‐cells act as effector‐memory T cells with glycolytic enhancement. In contrast, 4‐1‐BB‐based CAR T‐cells play a central memory T cells, relying on proper fatty acid metabolism.^14^, ^15^, ^16^ The distinctive role on CAR T‐cells makes the development of higher generation of CAR combining all these advantage to achieve better treatment outcomes. However, treatment strategies with either CAR T‐cells co‐expressing different co‐stimulatory domains or combining CAR T‐cells expressing different co‐stimulatory domain remains to be addressed in the future, which is critical for further precision translation. Interestingly, it has been shown that 4‐1‐BB but not CD28 co‐stimulatory element can activate noncanonical nuclear factor‐κB (NF‐kB) signaling.

Due to the small cohort size, we could not analyze the impact on survival incurred by co‐stimulatory domains. Though the sample size is too small to draw any conclusion with statistical significance, our preliminary data demonstrate that our anti‐CLL1‐based CAR T‐cells therapy is more suitable for allo‐HSCT bridging therapy but not a curable option. Meanwhile, we preliminarily demonstrated that the efficacy and safety of anti‐CLL1‐based CAR T‐cells in R/R‐AML treatment were unrelated to the co‐stimulatory elements. Indeed, a recent study by Li et al. has added more evidence to show the promising role of anti‐CLL1‐based CAR T‐cells (CD28/CD27 co‐stimulatory) in children with R/R‐AML, which is consistence with our previous report.^2^ To this end, data on the safety and efficacy of anti‐CLL1‐based CAR T‐cells on R/R‐AML remain very few, which should be addressed in a multicenter study in the near future.

## AUTHOR CONTRIBUTIONS

**Kunlin Pei:** Writing – original draft (equal); writing – review and editing (equal). **Haoyu xu:** Writing – original draft (equal). **Pengfei Wang:** Data curation (supporting). **Wenting Gan:** Data curation (supporting). **Zhengbin Hu:** Data curation (supporting). **Xiaoling Su:** Data curation (supporting). **Hui Zhang:** Investigation (lead); validation (lead); writing – review and editing (lead). **Yingyi He:** Resources (lead); writing – original draft (lead); writing – review and editing (equal).

## FUNDING INFORMATION

This work was partially supported by grants from National Natural Science Foundation of China (82170152) (H.Z).

## CONFLICT OF INTEREST STATEMENT

The authors had no potential conflicts of interest to disclose. All the authors have critically reviewed the manuscript and approved the final submission.

## ETHICS STATEMENT

The ethical approval (2018050201, 2018050202, 2018050803, and 2020–23) was obtained from the ethics committee at Guangzhou Women and Children's Medical Center.

## PATIENT CONSENT STATEMENT

Informed consent was completed by the parents and/or patients themselves if they were over 8 years old, according to the Declaration of Helsinki.

## CLINICAL TRIAL REGISTRATION NUMBERS

The study was registered at www.chictr.org.cn (ChiCTR1900027684, 4‐1‐BB) and https://clinicaltrials.gov/ (NCT03222674, CD28/CD27).

## Supporting information


**Data S1:** Supporting Information

## Data Availability

Not available.

## References

[1] Newell LF , Cook RJ . Advances in acute myeloid leukemia. BMJ. 2021;375:n2026. 34615640 10.1136/bmj.n2026

[2] Zhang H , Wang P , Li Z , He Y , Gan W , Jiang H . Anti‐CLL1 chimeric antigen receptor T‐cell therapy in children with relapsed/refractory acute myeloid leukemia. Clin Cancer Res. 2021;27:3549‐3555. 33832948 10.1158/1078-0432.CCR-20-4543

[3] Zhang H , Gan WT , Hao WG , Wang PF , Li ZY , Chang LJ . Successful anti‐CLL1 CAR T‐cell therapy in secondary acute myeloid leukemia. Front Oncol. 2020;10:685. 32528876 10.3389/fonc.2020.00685 PMC7266936

[4] Wang J , Chen S , Xiao W , et al. CAR‐T cells targeting CLL‐1 as an approach to treat acute myeloid leukemia. J Hematol Oncol. 2018;11:7. 29316944 10.1186/s13045-017-0553-5 PMC5761206

[5] Langebrake C , Brinkmann I , Teigler‐Schlegel A , et al. Immunophenotypic differences between diagnosis and relapse in childhood AML: implications for MRD monitoring. Cytometry B Clin Cytom. 2005;63:1‐9. 15624201 10.1002/cyto.b.20037

[6] Safarzadeh Kozani P , Safarzadeh Kozani P , Rahbarizadeh F . CAR‐T cell therapy in T‐cell malignancies: is success a low‐hanging fruit? Stem Cell Res Ther. 2021;12:527. 34620233 10.1186/s13287-021-02595-0 PMC8499460

[7] Roselli E , Boucher JC , Li G , et al. 4‐1BB and optimized CD28 co‐stimulation enhances function of human mono‐specific and bi‐specific third‐generation CAR T cells. J Immunother Cancer. 2021;9(10):e003354. 34706886 10.1136/jitc-2021-003354 PMC8552146

[8] Julamanee J , Terakura S , Umemura K , et al. Composite CD79A/CD40 co‐stimulatory endodomain enhances CD19CAR‐T cell proliferation and survival. Mol Ther. 2021;29:2677‐2690. 33940156 10.1016/j.ymthe.2021.04.038 PMC8417513

[9] Guercio M , Orlando D , Di Cecca S , et al. CD28.OX40 co‐stimulatory combination is associated with long in vivo persistence and high activity of CAR.CD30 T‐cells. Haematologica. 2021;106:987‐999. 32381575 10.3324/haematol.2019.231183 PMC8018158

[10] Hoechstetter MA , Busch R , Eichhorst B , et al. Prognostic model for newly diagnosed CLL patients in Binet stage a: results of the multicenter, prospective CLL1 trial of the German CLL study group. Leukemia. 2020;34:1038‐1051. 32042081 10.1038/s41375-020-0727-y

[11] Pollyea DA , Bixby D , Perl A , et al. NCCN guidelines insights: acute myeloid leukemia, version 2.2021. J Natl Compr Canc Netw. 2021;19:16‐27. 33406488 10.6004/jnccn.2021.0002

[12] Mahadeo KM , Khazal SJ , Abdel‐Azim H , et al. Management guidelines for paediatric patients receiving chimeric antigen receptor T cell therapy. Nat Rev Clin Oncol. 2019;16:45‐63. 30082906 10.1038/s41571-018-0075-2 PMC7096894

[13] Santomasso BD , Nastoupil LJ , Adkins S , et al. Management of immune‐related adverse events in patients treated with chimeric antigen receptor T‐cell therapy: ASCO guideline. Clin Oncol. 2021;39:3978‐3992. 10.1200/JCO.21.01992 34724386

[14] van der Stegen SJ , Hamieh M , Sadelain M . The pharmacology of second‐generation chimeric antigen receptors. Nat Rev Drug Discov. 2015;14:499‐509. 26129802 10.1038/nrd4597 PMC6410718

[15] Porter DL , Hwang WT , Frey NV , et al. Chimeric antigen receptor T cells persist and induce sustained remissions in relapsed refractory chronic lymphocytic leukemia. Sci Transl Med. 2015;7:303ra139. 10.1126/scitranslmed.aac5415 PMC5909068 26333935

[16] Kawalekar OU , O'Connor RS , Fraietta JA , et al. Distinct signaling of coreceptors regulates specific metabolism pathways and impacts memory development in CAR T cells. Immunity. 2016;44:380‐390. 26885860 10.1016/j.immuni.2016.01.021 PMC13498396

[17] Griffin JD . Leukemia stem cells and constitutive activation of NF‐kappaB. Blood. 2001;98:2291. 10.1182/blood.v98.8.2291a 11588020

