# Supplementary material for: Anti‐CLL1‐based CAR T‐cells with 4‐1‐BB or CD28/CD27 stimulatory domains in treating childhood refractory/relapsed acute myeloid leukemia
Source: Cancer Med. 2023 Apr 9;12(8):9655–61. doi: 10.1002/cam4.5916 (PMC10166968; doi:10.1002/cam4.5916)
Supplement: Supplementary file 1 — Data S1: Supporting Information [file CAM4-12-9655-s001.pdf]

Supplementary materials

Study design

Study title

A phase I/II single-arm, single dose clinical trial to evaluate the safety and preliminary efficacy of CLL1 CAR-T cell for relapsed/refractory pediatric acute myeloid leukemia.

Subject age

1-18 years old (containing boundary value).

Dosage

Single dose, 1E6/kg±20%, intravenous infusion.

Primary study objective

To evaluate the safety and tolerance of CLL1 CAR-T infusion in subjects with relapsed/refractory acute myeloid leukemia (R/R AML).

Secondary study objectives

(1) To evaluate the pharmacokinetic (PK) properties of CLL1 CAR-T infusion in r/r AML subjects;
(2) To evaluate the initial efficacy of CLL1 CAR-T infusion in r/r AML subjects defined as overall response rate (ORR) and composite CR (CRc) rate;
(3) To evaluate rate of negative measurable residual disease (MRD);
(4) To evaluate the overall survival (OS), event-free survival (EFS), relapse-free survival (RFS) of CLL1 CAR-T infusion in r/r AML subjects;
(5) To evaluate the immunogenicity of CLL1 CAR-T infusion in r/r AML subjects.

Exploratory objective

To explore changes in vivo cytokine profile before and after infusion of CLL1 CAR-T in r/r AML subjects.

Inclusion criteria

1. The subject voluntarily signs the informed consent form and is expected to complete study processes including follow-up and treatment;
2. 1-18 years old (containing boundary value);
3. Confirmed AML per WHO 2016 criteria with relapsed and/or refractory disease:
    a) Diagnostic criteria for relapsed AML: reappearance of leukemia blasts in the peripheral blood or in bone marrow≥5% (excluding other reasons such as bone marrow regeneration after consolidation chemotherapy) or extramedullary infiltration of leukemic cells after achievement of CR/CRi/MLFS;
    b) Diagnostic criteria for refractory AML: failure to achieve CR/CRi/MLFS, or with persistent extramedullary leukemia after 1 course of purine analogue containing induction chemotherapy (such as FLAG-Ida, CLIA, or other similar regimens) or 2 courses of 3+7 based intensive chemotherapy-based treatment or with 3 cycles of HMA-based lower intensity therapies including venetoclax containing lower intensity regimens;
    c) The patient has recovered from the toxicity of previous treatment, i.e. CTCAE toxicity grading scale <2 (unless the abnormalities are related to tumor).
4. ECOG scale of performance status 0～1 and expected survival over 3 months;
5. Patients with proper organ functions:
    - Aspartate Transaminase (AST) ≤ 3 times the upper limit of normal (ULN);
    - Alanine aminotransferase (ALT) ≤ 3 times ULN;
    - Total bilirubin ≤ 2 times ULN (unless due to Gilbert’s s);
    - Serum creatinine ≤ 1.5 times ULN, or creatinine clearance rate ≥ 50 mL/min assessed using Cockcroft-Gault formula or 24-hour urine collection;
    - Hemoglobin ≥ 70 g/L or hemoglobin is maintained at this level after blood transfusion;
    - Left ventricular ejection fraction (LVEF) ≥ 45%.
6. Patents with relapse after allogeneic stem cell transplant (allo-SCT) will be eligible

after 6 months from date of prior transplant and will need to have recovered from all transplant-related complications and need to be off of all immunosuppression for ≥6 weeks, with no more than grade 1 chronic graft-versus host disease (cGVHD);

7. Female subjects must also meet the following criteria before enrollment can be considered:
   a Infertility, defined as:
   - Have undergone hysterectomy or bilateral oophorectomy, or
   - Have received bilateral tubal ligation, or
   - Postmenopausal (absolute amenorrhea ≥ 1 year).

   b Fertile but tested negative for serum pregnancy test at screening, and agree to use medically approved contraceptives (such as IUD, contraceptive drugs or condoms) prior to study enrollment and during the study until 6 months after the last administration of the study drug.
8. Sexually active male patients must agree to use barrier contraceptives or adopt total abstinence.

<u>Exclusion criteria</u>

1. Patients with acute promyelocytic leukemia (APL);
2. Evidence of central nervous system (CNS) involvement or craniocerebral neuropathy due to leukemia; prior CNS pathology within 6 months of screening including epilepsy requiring anti-epileptic medication, brain injury, organic brain disorder; severe psychiatric disorder;
3. Prior therapy with CLL-1 targeting CAR-T or antibody drug conjugate (may be permitted after discussion with sponsorsif deemed to have inadequate therapy);
4. Known positive for hepatitis B surface antigen (HBsAg) or hepatitis B core antibody (HBcAb) or hepatitis C virus (HCV) antibody. Patients may be eligible if treated with antiviral therapy and have had undetectable viral DNA testing for 6 months;

5. Known positive for human immunodeficiency virus (HIV) antibody;
6. Known positive for cytomegalovirus (CMV) DNA test;
7. Known allergic reactions to any of the ingredients used in the study treatment;
8. Severe and/or uncontrolled heart disease, including but not limited to severe arrhythmia, unstable angina pectoris, myocardial infarction within 6 months, New York Heart Association (NYHA) class III or IV heart failure, and refractory hypertension (refractory hypertension is defined as: abnormal blood pressure after treatment with reasonable, tolerable and adequate doses of ≥3 anti-hypertension drugs (including diuretics) on top of improved lifestyle for >1 month or blood pressure effectively controlled after administration of ≥ 4 anti-hypertension drugs); significant pulmonary dysfunction; uncontrolled venous or arterial thromboembolism;
9. Previously received or prepares to receive organ transplantation (except hematopoietic stem cell transplantation);
10. Acute GVHD or chronic GVHD grade >1;
11. Received hematopoietic stem cell transplantation within 6 weeks prior to screening;
12. Active autoimmune or inflammatory disorders of the nervous system (e.g. Guillain-Barre Syndrome (GBS), amyotrophic lateral sclerosis (ALS)) and clinically significant active cerebrovascular diseases (e.g. cerebral edema, posterior reversible encephalopathy syndrome (PRES));
13. Patients with concurrent active malignancy requiring continuous or intermittent active antineoplastic or radiation therapy. Patients with skin cancers or localized or in situ cancers which have been removed or definitively treated, or patients on maintenance therapy or endocrine therapy for well-controlled malignancy may be allowed after discussion with sponsors;
14. Patients with contraindications to any study procedure or other medical conditions that may expose them to unacceptable risks in accordance with the investigator's judgment and/or clinical criteria;
15. Women who are currently pregnant or breast-feeding.

Treatment response evaluation

Bone marrow morphologic and flow cytometric assessments for treatment response are performed every month for the first three months after CAR-T cell therapy, and every three months thereafter if not followed by allo-HSCT. The European LeukemiaNet (ELN) recommendations for diagnosis and management of AML (2017) are used to evaluate the treatment response in this study.

(1) Complete remission (CR)

- <5% blasts in the bone marrow;
- No blasts with Auer rods;
- Normal maturation of all cellular components in the bone marrow;
- No extramedullary disease;
- Neutrophils ≥ 1000/μL; platelets ≥ 100,000/μL;
- Transfusion independent.

(2) Complete remission with incomplete hematologic recovery (CRi)

- Meets all CR criteria except for residual neutropenia or thrombocytopenia (neutrophils < 1000/μL or platelets < 100,000/μL).

(3) Morphologic leukemia-free state (MLFS)

- Bone marrow blasts <5%;
- Absence of blasts with Auer rods;
- Absence of extramedullary disease;
- No hematologic recovery required.

(4) Partial remission (PR)

- Meets all hematologic criteria of CR, but with decrease of bone marrow blast percentage to 5% - 25% and decrease of pretreatment bone marrow blast percentage by at least 50%.

(5) Stable disease (SD)

- Absence of CR, CRi, MLFS, PR, and does not meet the criteria for PD.

(6) Progressive disease (PD)

- Evidence for an increase in bone marrow blast percentage and/or increase of

absolute blast counts in the blood:

- >50% increase in marrow blasts over baseline (a minimum 15% point increase is required in cases with <30% blasts at baseline); or persistent marrow blast percentage >70% over at least 3 months; without at least a 100% improvement in ANC to an absolute level >0.5 x 10^9/L, and/or platelet count to >50 x 10^9/L in non-transfused condition;
- Or >50% increase in peripheral blasts (WBC x % blasts) to >25 x 10^9/L (in the absence of differentiation syndrome);
- Or with new extramedullary disease.

(7) Relapse

- After CR, bone marrow blasts >5%;
- Or reappearance of blasts in the blood;
- Or development of extramedullary disease.

Study cycles and study processes

The overall trial process is as follows:

Screening period (D-25~D-19): the informed consent form is signed, the subjects are screened based on the inclusion criteria, baseline demographic data and disease status are recorded.

⬇

Blood sampling period (D-18~D-15): leukapheresis is performed on the subjects to obtain T cells needed for CAR-T cell production.

⬇

Pre-treatment period (D-14~D-2): CAR-T cells are produced during this period and the inclusion/exclusion criteria are reexamined. Subjects who satisfy the inclusion criteria need to undergo pretreatment of lymphodepleting chemotherapy (LD chemotherapy) in preparation for CAR-T cell infusion. The subjects need to be evaluated based on the eligibility criteria for lymphodepletion prior to lymphodepleting chemotherapy.

⬇

Pre-reinfusion evaluation (D-1): Pre-reinfusion evaluation will be performed, laboratory

test results and physical characteristics of the patients will be recorded.

↓

Infusion period (D0): intravenous infusion of CAR-T cells will be performed according to the methods of use and dosage specified in the protocol.

↓

Observation period (D1~D28): hospitalization will be recommended from D1 to D14 for observation, per the treating physicians discretion, followed by follow-up visits on D21 and D28 when safety, efficacy and other associated parameters will be tracked.

↓

Follow-up period (D29~M24): according to the follow-up plan, the subjects will come to the hospital for safety checks, efficacy checks and other related checks on M2, M3, M6, M9, M12, M18 and M24.

Supplementary Figure 1

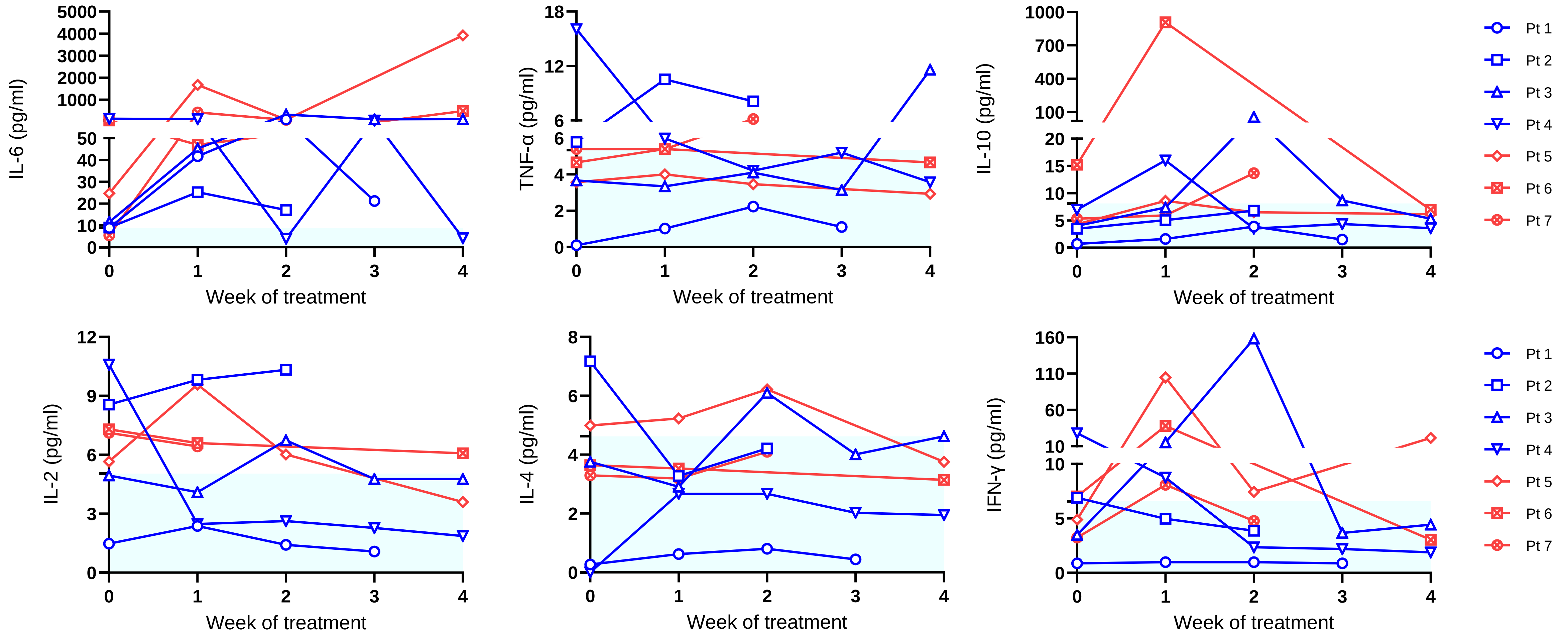

Supplementary Figure 1. Cytokine level during anti-CLL1-based CAR T-cells treatment. The light blue shade represents the normal reference value.

Supplementary Figure 2

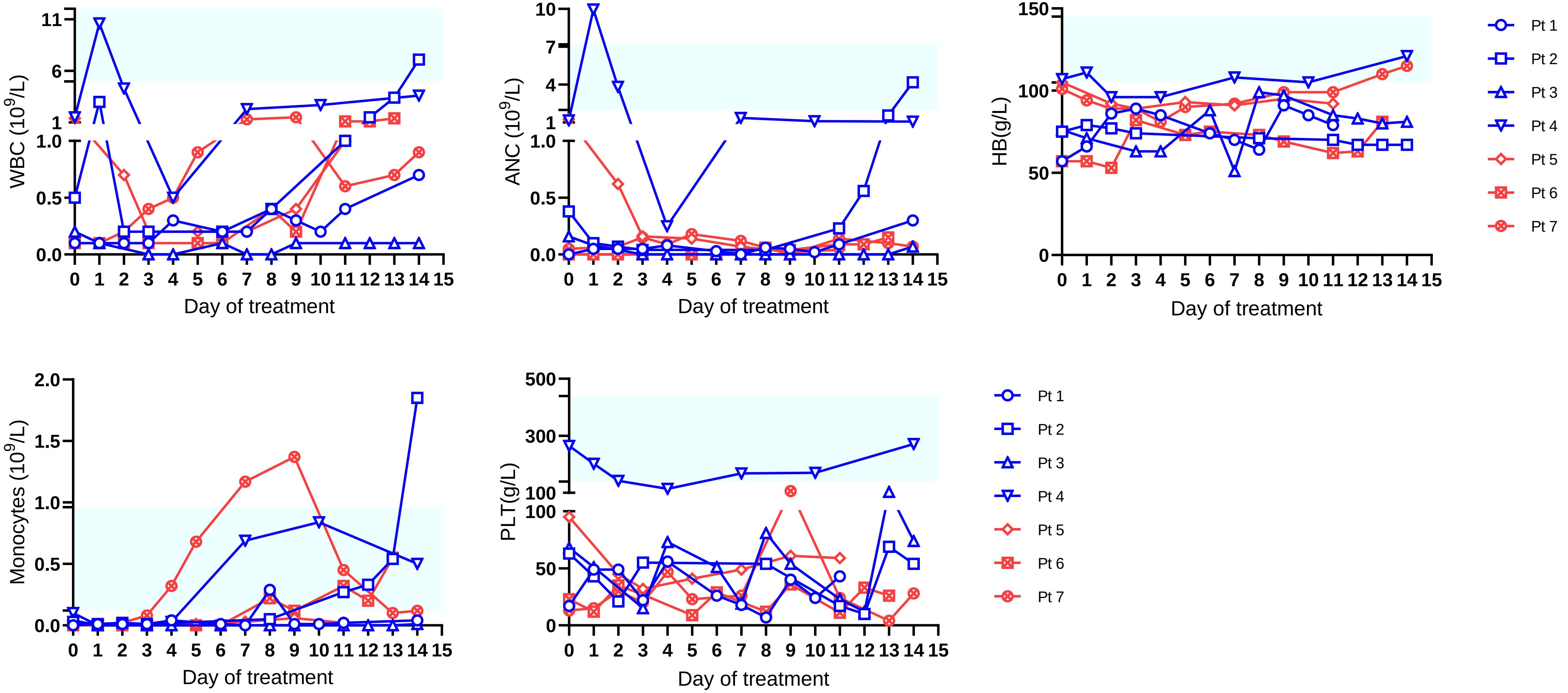

Supplementary Figure 2. Blood cell count index during anti-CLL1-based CAR T-cells treatment. The light blue shade represents the normal reference value.

Supplementary Figure 3

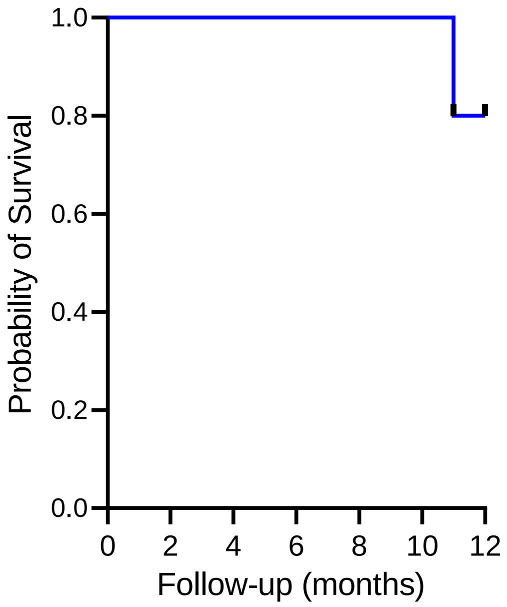

Supplementary Figure 3. The 1-yr overall survival among these five responded patients.
